# Research on the Pavement Performance of Slag/Fly Ash-Based Geopolymer-Stabilized Soil

**DOI:** 10.3390/ma18133173

**Published:** 2025-07-04

**Authors:** Chenyang Yang, Yan Jiang, Zhiyun Li, Yibin Huang, Jinchao Yue

**Affiliations:** 1 School of Water Conservancy and Transportation, Zhengzhou University, Zhengzhou 450001, China; 18530951762@163.com (C.Y.); benbenjiangyan@163.com (Y.J.); yuejc@zzu.edu.cn (J.Y.); 2 Geotechnical Engineering Research Institute, Guangdong Research Institute of Water Resources and Hydropower, Guangzhou 510610, China; lizhiy29@163.com

**Keywords:** slag-based geopolymer, fly ash, stabilized soil, semi-rigid base, compressive strength, pavement performance, freeze–thaw resistance

## Abstract

The road construction sector urgently requires environmentally friendly, low-carbon, and high-performance base materials. Traditional materials exhibit issues of high energy consumption and carbon emissions, making it difficult for them to align with sustainable development requirements. While slag- and fly ash-based geopolymers demonstrate promising application potential in civil engineering, research on their application in road-stabilized soils remains insufficient. To address the high energy consumption and carbon emissions associated with conventional road base materials and to fill this research gap, this study investigated the utilization of industrial solid wastes through slag-based geopolymer and fly ash as stabilizers, systematically evaluating the pavement performance of two distinct soil types. Unconfined compressive strength tests and freeze–thaw cycling tests were conducted to elucidate the effects of stabilizer dosage, fly ash co-stabilization, and compaction degree on mechanical properties. The results demonstrated that the compressive strength of both stabilized soils increased significantly with higher slag-based geopolymer content, achieving peak values of 5.2 MPa (soil sample 1) and 4.5 MPa (soil sample 2), representing a 30% improvement over cement-stabilized soils with identical mix proportions. Fly ash co-stabilization exhibited more pronounced reinforcement effects on soil sample 2. At a 98% compaction degree, soil sample 1 maintained a stable 50% strength enhancement, whereas soil sample 2 displayed a dose-dependent exponential strength increase. Freeze–thaw resistance tests revealed the superior performance of soil sample 1, showing a loss of compressive strength (BDR) of 78% with 8% geopolymer stabilization alone, which improved to 90% after fly ash co-stabilization. For soil sample 2, the BDR increased from 64% to 80% through composite stabilization. This study confirms that slag/fly ash-based geopolymer-stabilized soils not only meet the strength requirements for heavy-traffic subbases and light-traffic base courses, but also demonstrates its great potential as a low-carbon and environmentally friendly material to replace traditional road base materials.

## 1. Introduction

In recent years, the continuous expansion of infrastructure construction has led to the extensive utilization of traditional cementitious materials (e.g., Portland cement, lime), causing significant issues, including high energy consumption, environmental pollution, and excessive resource extraction [1,2]. The road construction sector urgently requires environmentally friendly alternatives to replace traditional base materials characterized by high energy consumption and carbon emissions. Slag- and fly ash-based geopolymers, owing to their high strength, excellent durability, and environmental friendliness, have been widely adopted in civil engineering. The application of such materials in soil stabilization for roads can not only effectively mitigate the environmental impacts of traditional materials and utilize industrial solid waste, thereby reducing project costs, but also significantly enhance the performance of the base course. This holds considerable practical significance for advancing the sustainable development of road construction. Geopolymer materials are being applied in road base materials and building materials, and they have good performance [3,4]. Geopolymer materials are formed through the alkali-activated polycondensation of aluminosilicate precursors (fly ash, slag, etc.) into three-dimensional aluminosilicate networks [5,6]. Compared to traditional ordinary Portland cement, geopolymers exhibit superior strength, low CO_2_ emissions during production, and excellent durability [7,8,9], offering a novel alternative to conventional cementitious materials. Current high-grade highway construction predominantly employs cement-stabilized semi-rigid base courses, which are prone to thermal shrinkage and desiccation cracks, leading to base loosening and pumping phenomena [10,11], severely compromising pavement service life and traffic safety. With the intensified coupling effects of traffic loads and environmental exposure, enhanced requirements have been imposed on the bearing capacity and durability of base course materials. Against this background, the development of geopolymer-based soil stabilization technology not only facilitates industrial solid waste recycling but also improves the crack resistance and durability of base courses, demonstrating strategic significance for advancing green transition in highway construction.

Internationally, extensive research has been conducted on geopolymer applications in road base and subbase courses, primarily focusing on the recycling of industrial waste materials, treatment of unfavorable geological conditions, and recycled concrete base layers. Hanegbi et al. [12] investigated the stabilization of silt loam soil with an MK-based geopolymer. It was found that with the use of the geopolymer, the tensile load resistance of improved soil increased up to 6 kN, and the dust emission significantly decreased. Hou et al. [13] and Wang et al. [14] investigated cement-stabilized aggregate base courses, revealing that while such bases exhibit higher strength compared to non-cementitious stabilized aggregates and enhance the overall performance of flexible pavements, the cement hydration process may induce shrinkage cracks. These cracks can propagate as reflective cracks in asphalt surface layers, ultimately compromising the integrity of flexible pavement systems. As a specialized novel cementitious material, geopolymers demonstrate the potential to replace ordinary Portland cement as stabilizers for aggregate bases. Yue et al. [15] investigated the effects of the curing period and dosage of slag-based geopolymer on the mechanical properties, frost resistance, and drying shrinkage rate of geopolymer-stabilized crushed rock. The results demonstrated that with increasing geopolymer dosage, both the mechanical performance and frost resistance of the stabilized aggregate were significantly enhanced. Furthermore, slag–fly ash geopolymer-stabilized crushed rock exhibited superior drying shrinkage resistance, compared to cement–fly ash stabilized counterparts. Synthesized from aluminosilicate materials and alkaline activators, geopolymers undergo a sequential reaction mechanism in highly alkaline environments: dissolution of aluminosilicate components, polymerization of dissolved minerals, precipitation of hydrated products, and final hardening of inorganic polymer matrices. This process enables the effective valorization of industrial solid wastes, including red mud, fly ash, steel slag, and blast furnace slag.

Current research findings indicate that geopolymer production may generate only one-fifth to half of the CO_2_ emissions, compared to ordinary Portland cement production [16]. Furthermore, geopolymers exhibit characteristics such as high strength, low shrinkage, and acid resistance when compared with ordinary Portland cement, making them particularly suitable as a potential cementitious material for stabilized aggregate base courses in pavement structures. However, most previous studies have been limited to characterizing geopolymer pastes, mortars, and concrete, while overlooking the potential of utilizing geopolymers as stabilizing agents for pavement base materials. Hu et al. [17] and Wang et al. [18] investigated the feasibility of utilizing fly ash and red mud to produce geopolymers as cementitious materials for crushed stone aggregates. Laboratory measurements were conducted to evaluate the unconfined compressive strength, failure strain, and drying shrinkage of geopolymer-stabilized aggregate specimens under varying conditions. A comparative analysis was performed against the physical–mechanical properties of conventional stabilized base materials commonly used in pavement engineering. The results demonstrated that environmental humidity and temperature exhibited pronounced effects on strength development, while the dry shrinkage strain was slightly lower than that of traditional stabilized base materials. Extensive research has been conducted by scholars both domestically and internationally on the application of geopolymers in construction. For instance, Nawaz et al. [19] discussed recent advancements in using geopolymers as construction materials within civil engineering applications. The results demonstrated that geopolymers can achieve performance comparable to traditional concrete in terms of shear strength and durability while simultaneously reducing environmental impact. In another study, Terrones-Saeta et al. [20] prepared geopolymers using tailings from the Linares lead mine as raw material, activated chemically using potassium hydroxide. To this end, the study tested varying proportions of alkaline activators and evaluated the physical and mechanical properties of the formed materials. The findings indicated that the performance of the produced geopolymer materials met the requirements for construction materials. Sharmin et al. [21] reviewed the recent advancements of the integration of clay brick wastes in geopolymer applications, individually as well as its use with other alternative materials, expanding the potential uses of clay brick waste in areas such as waste management, soil stabilization, or alternative building materials.

Arulrajah et al. [22] investigated the mechanical properties of geopolymer-stabilized recycled construction materials for potential application in road base or subbase courses. Three precursor materials—fly ash (FA), slag (S), and carbide lime residue (CCR)—were employed to formulate geopolymers for stabilizing recycled concrete aggregates (RCA) and crushed bricks. Through unconfined compressive strength and resilient modulus testing, the study demonstrated that the combination of CCR with 5% slag (CCR + 5%S) exhibited the most favorable mechanical performance in stabilizing construction and demolition waste materials. Ahirwar et al. [23] and Khodair et al. [24] conducted a study to fully utilize construction and demolition waste by developing recycled aggregate concrete through the complete replacement of natural aggregates with construction and demolition waste aggregates, combined with fly ash and alkali activators. Mechanical strength testing revealed that the 7-day compressive strength and splitting tensile strength of fly ash-based geopolymer recycled concrete were 6–15% lower, compared to ordinary Portland cement concrete. In a related study, Suksiripattanapong et al. [9] applied fly ash-based geopolymers to stabilize sewage treatment sludge for the development of geotechnical blocks. Experimental validation demonstrated that geopolymer-stabilized sludge blocks exhibited superior strength and durability over cement-stabilized sludge blocks. Shen et al. [25] developed a novel binder composed of steel slag, fly ash, and phosphogypsum for potential application in pavement base courses. The optimal mix proportion was determined as a fly ash-to-steel slag ratio of 1:1, with 2.5% phosphogypsum content. Experimental evaluations demonstrated that the stabilized base material exhibited superior water stability compared to lime–fly ash-stabilized soil and higher long-term strength than cement-stabilized granular materials, satisfying all specifications for pavement base applications. The study further elucidated the strength formation mechanisms through microstructural and chemical analyses.

Hayder et al. [26] investigated the effects of different amounts of MK addition on the soil’s mechanical properties. Furthermore, the effects of parameters, such as the type and concentration of the alkaline solution and curing time, on the unconfined compressive strength, failure strain, Young’s modulus, California bearing ratio, and direct shear test were evaluated. The results indicated that the soil samples that were stabilized with MK 10% and NaOH had a notably higher compressive strength (2936 kPa), indicating a denser and less porous structure (improved stiffness stabilized soil) in comparison to the soil samples stabilized with MK 10% and Na_2_SiO_3_, which was (447 kPa). Li et al. [27] studied the effect of alkali dosage on the mechanical properties and water resistance of alkali-activated brick powder geopolymers. The results showed that at a low alkali dosage, the geopolymer of brick powder had a loose microstructure and poor compressive and flexural strengths. At high dosages, a denser microstructure was produced, thereby improving mechanical properties. Teerawattanasuk et al. [28] conducted a comparative study on the stabilization effects of cement and alkali-activated fly ash geopolymer on lateritic soils. Field CBR (California bearing ratio) testing confirmed compliance with relevant specifications for both stabilization methods, with predictive equations correlating curing age with CBR values being established. Compared to cement-stabilized soils, the geopolymer-stabilized soils demonstrated markedly superior early-age strength characteristics. Microstructural analysis revealed that stabilized soil specimens with higher CBR values exhibited more compact microstructures, substantiating the feasibility of employing fly ash geopolymers as stabilizing agents for pavement subbase materials.

Current research on the pavement performance of slag/fly ash-based geopolymer-stabilized soils is limited, and further studies are required. This study used geopolymer as a stabilizer to modify two soils with different properties. Experimental investigations were conducted, mainly including the effects of slag-based geopolymer dosage on the mechanical strength and freeze–thaw resistance of stabilized soils, as well as comparisons with the characteristics of cement-stabilized soils. The aim was to provide a theoretical basis and practical references for the application of slag/fly ash-based geopolymers in road engineering.

## 2. Materials and Methods

### 2.1. Materials

**Figure 1:**
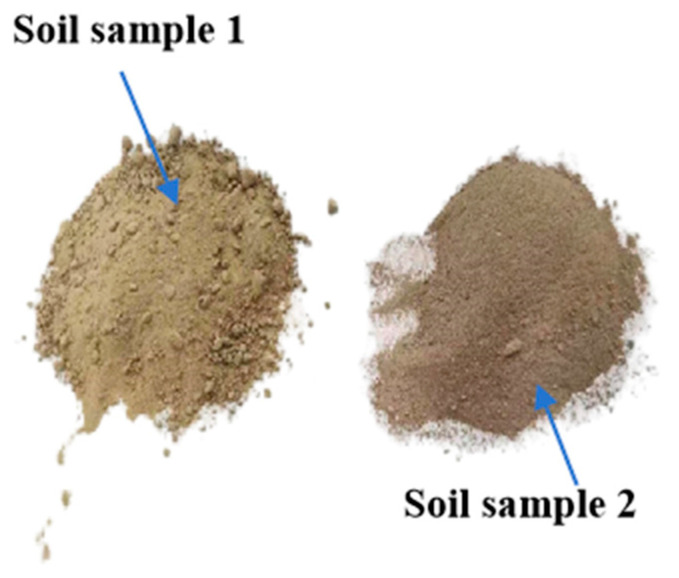
Soil sample 1 (**left**) and soil sample 2 (**right**).

The two soil specimens utilized in this study were sourced from a road construction site in Pingdingshan and the experimental field station of Zhengzhou University, as illustrated in Figure 1. Therefore, the region most relevant to this study was Henan Province, China. The liquid limit and plastic limit test results are summarized in Table 1. The data indicate that soil sample 1 exhibited a high plasticity index of 17, while soil sample 2 demonstrated a lower plasticity index of 7.9. Following collection, the soils underwent standardized preparation procedures, including oven-drying, mechanical crushing, and sieving to remove oversized particles, resulting in two soil types with comparable particle size distributions.

**Figure 2:**
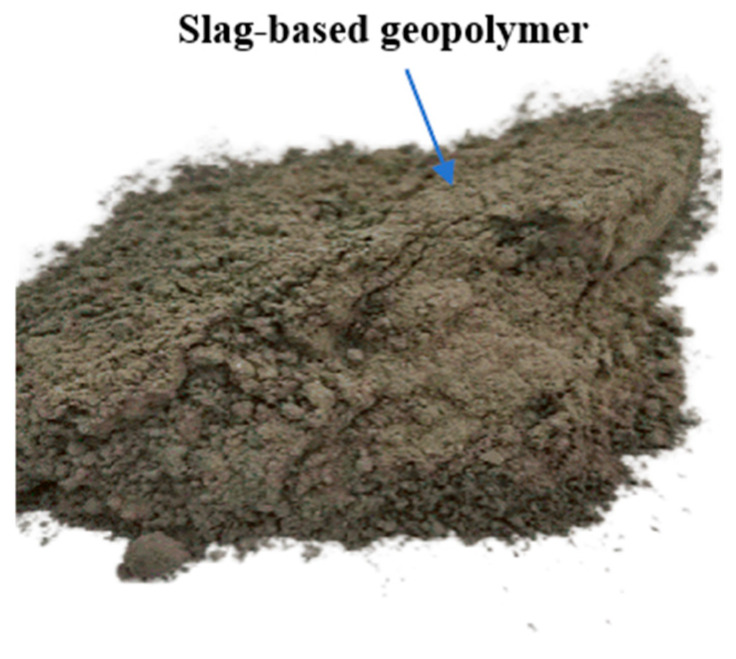
Slag-based geopolymer material.

The slag-based geopolymer material used in the experiments, as shown in Figure 2, was composed of blast furnace slag, alkaline activator, and other components. Slag, a byproduct discharged from blast furnaces during pig iron production, contains abundant amorphous aluminosilicate complexes and exhibits cementitious hydraulic activity similar to Portland cement. The main chemical compositions are listed in Table 2. The S95-grade slag utilized in this study was sourced from Gongyi Water Purification Co., Ltd. (Gongyi, China), with its primary chemical composition detailed in Table 2. The alkaline activator was formulated by blending sodium silicate and NaOH to provide the alkaline environment required for geopolymer reactions.

The fly ash employed was Class F Grade I fly ash, obtained from Borun Refractory Materials Co., Ltd. (Gongyi, China), with its chemical composition also listed in Table 2. The cement used was 42.5-grade ordinary Portland cement from Zhengzhou Jinlong Cement Plant (Zhengzhou, China), and its chemical composition is similarly provided in Table 2.

### 2.2. Mix Ratios of Slag-Based Geopolymer-Stabilized Soils

In the compaction text, a total of 36 specimens were prepared. The types of soil samples were divided into soil sample 1 and soil sample 2, and the stabilizers were classified as single-admixture geopolymer, geopolymer, and fly ash compound admixture. The dosages of single geopolymer were 4%, 6%, and 8%. When mixed, the mass ratios of geopolymer, fly ash, and soil were set as 4:8:88, 6:12:82, and 8:16:76, respectively.

In the unconfined compressive strength test, a total of 324 specimens were prepared. The types of soil samples were divided into soil sample 1 and soil sample 2. The stabilizers were classified as single-mixed geopolymer, single-mixed cement, and separately mixed with fly ash. The dosages of geopolymer and cement alone were 4%, 6%, and 8%, respectively. When mixed, the mass ratios of geopolymer or cement, fly ash, and soil were set as 4:8:88, 6:12:82, and 8:16:76, respectively. The curing ages were divided into 7 days, 14 days, and 28 days. The compaction degree was divided into 89% and 98%, and when cement was used as a stabilizer, the compaction degree was only set at 98%.

In the freeze–thaw test, a total of 72 specimens were prepared. The types of soil samples were divided into soil sample 1 and soil sample 2. The stabilizers were classified as single-mixed geopolymer, single-mixed cement, and separately mixed with fly ash. The dosages of geopolymer and cement alone were 4%, 6%, and 8%, respectively. When mixed, the mass ratios of geopolymer or cement, fly ash, and soil were set as 4:8:88, 6:12:82, and 8:16:76, respectively. The curing age was 14 days. The compaction degree was 98%.

### 2.3. Specimen Preparation

Specimens were prepared according to the optimum water content and maximum dry density. The optimal moisture content and the maximum dry density are shown in Table 3. Each material was weighed, pre-wetted, and cured. The curing time was 24 h, with a temperature ranging from 20 °C to 30 °C and a relative humidity above 80%. Then, stabilizers were added and mixed uniformly. Small cylindrical specimens (150 mm × φ150 mm) were formed using the static compaction method with compaction degrees of 98% and 89%. The static compaction method is a forming technique that uses a press to slowly, steadily, and continuously apply a constant pressure to the material in a mold and maintain the pressure, thereby compacting it to the target density and specified shape. After demolding, specimens were weighed, measured for height, sealed in plastic bags, and placed in a standard curing room. The standard curing room was maintained at a temperature of 20 ± 2 °C and a relative humidity of 95% or higher.

### 2.4. Testing Methods

#### 2.4.1. Compaction Test

Compaction tests were performed on stabilized soil mixtures, with mix ratios specified in Section 2.2, to determine the optimum moisture content and maximum dry density for each formulation. The compaction mold had an internal diameter of 10 cm and height of 12.7 cm. After homogenizing the mixture, it was compacted in three equal layers, each subjected to 27 blows. Upon completion, the specimen surfaces were trimmed with a soil knife and straightedge to ensure planar top and bottom interfaces. The total mass was recorded before demolding. The extracted specimen was mechanically disintegrated, and a representative subsample (approximately 100 g) was oven-dried at 110 °C for moisture content determination.

#### 2.4.2. Unconfined Compressive Strength Test

Unconfined compressive strength tests were conducted at curing ages of 7 d, 14 d, and 28 d. One day prior to the specified curing duration, specimens were removed and immersed in a 20 °C constant-temperature water tank for 24 h, with the water level maintained 2.5 cm above the specimen top. After immersion, specimens were surface-dried and subjected to compression testing using a pavement material strength tester. To ensure data accuracy, nine parallel tests were performed per group. Outliers were excluded, and representative strength values were calculated with a 95% assurance rate based on the remaining valid data. The compressive strength was determined by Formulas (1) and (2), where *R_c_* is the unconfined compressive strength; *P* is the peak failure load of samples; *A* is the cross-sectional area of samples; and *D* is the diameter of the test piece.

(1) $$R_{c}=\frac{P}{A}$$

(2) $$A=0{.25\pi D}^{2}$$

#### 2.4.3. Freeze–Thaw Cycle Test

Cement-stabilized soil is widely used in base and subbase courses of various classes of highways in seasonally frozen regions of northern China. As a common subgrade treatment material, it offers advantages, including high bearing capacity, substantial strength, rapid construction with machinery, and low cost [29]. However, highways in northern regions undergo annual freeze–thaw cycles, which induce distresses that not only reduce the pavement service life but also compromise the load-bearing capacity and stability of the pavement structure [30,31]. The base course, serving as the primary load-bearing component, must withstand stresses transferred from the surface layer and therefore requires high strength and stiffness.

**Figure 3:**
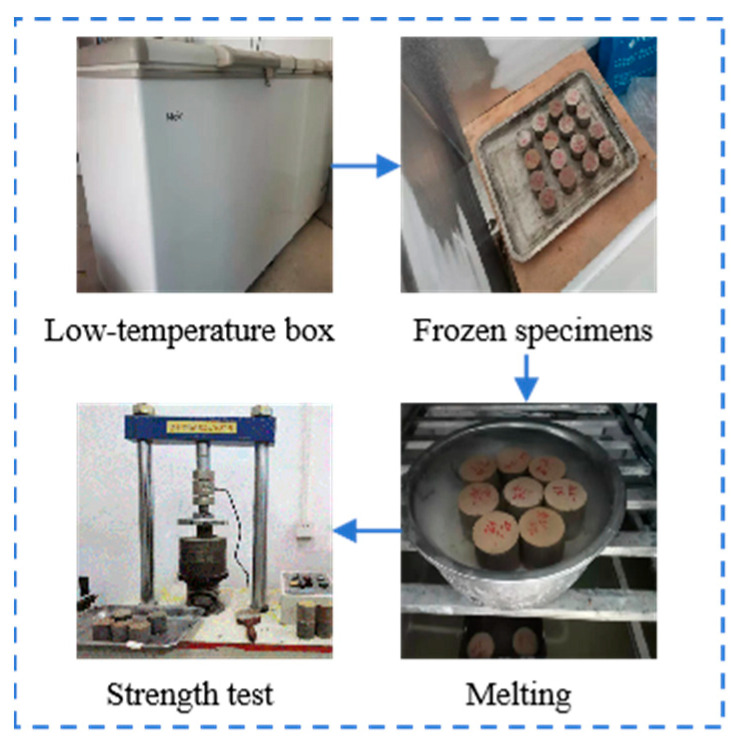
The test process.

This test adopted 14-day cured specimens subjected to 5 freeze–thaw cycles as the execution standard. The low-temperature chamber was set at −18 °C with 20 °C water immersion. Each freezing phase lasted 16 h, followed by an 8 h thawing period. Twelve standard cylindrical specimens (50 mm × φ50 mm) were prepared for each mix ratio: 6 for freeze–thaw testing and 6 as control specimens. On the final day of curing, specimens were water-saturated. After removal, surface moisture was wiped off, and specimens were weighed. Subsequent weighing was conducted after each freeze–thaw cycle, followed by compressive strength measurement. The test process is shown in Figure 3. The compressive strength loss (BDR) was determined by Formula (3), where *BDR* is the loss of compressive strength after n cycles of freeze–thaw; *R_DC_* is the compressive strength of the test piece after n cycles of freeze–thaw; and *R_C_* is the compressive strength of the control test piece.

(3) $$\mathrm{BDR}=\frac{R_{\mathrm{DC}}}{R_{C}}\times 100\%$$

## 3. Results and Discussion

### 3.1. Compaction Test

**Figure 4:**
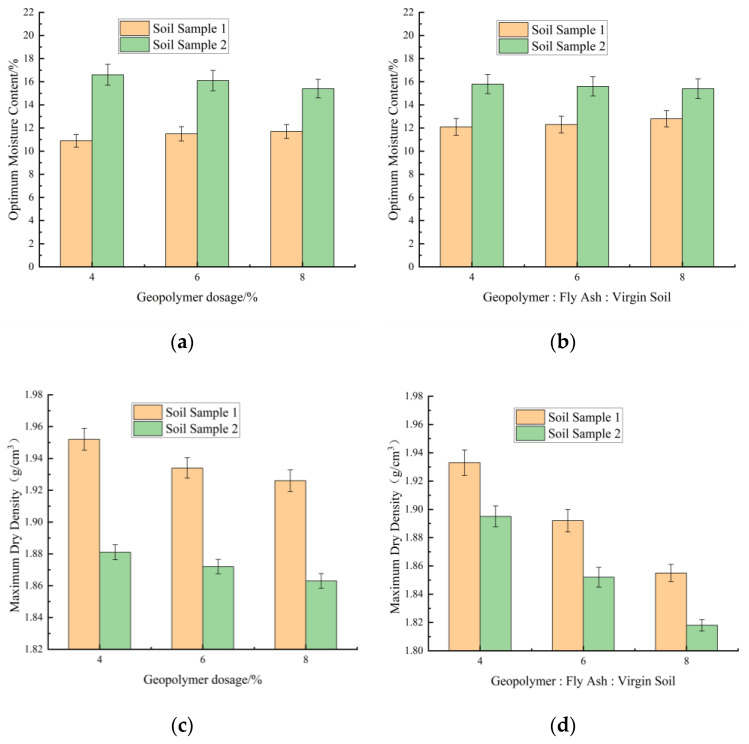
Variations of optimum moisture content and maximum dry density of stabilized soils with stabilizer dosage. (**a**) Variations of optimum moisture content with geopolymer dosage. (**b**) Variations of optimum moisture content with fly ash content. (**c**) Variations of maximum dry density with geopolymer dosage. (**d**) Variations of maximum dry density with fly ash content.

The variations of optimum moisture content and maximum dry density of stabilized soils with stabilizer dosage are presented in Figure 4a–d.

Overall, soil sample 1 exhibited a lower optimum moisture content compared to soil sample 2, while its maximum dry density was higher. The higher liquidity index and stronger water absorption capacity of soil sample 2 resulted in greater water demand for uniform wetting, consequently leading to a relatively lower density. As shown in Figure 4a,b, the optimum moisture content of soil sample 1 gradually increased with stabilizer content, whereas the opposite trend was observed for soil sample 2. This divergence primarily arose from the lower plasticity index and liquid limit of soil sample 1, whose water absorption rate was inferior to that of geopolymer and fly ash. Consequently, higher stabilizer content induced a slight optimum moisture content increment in soil sample 1. In contrast, the geopolymer and fly ash reduced the plasticity index of soil sample 2. As shown in Figure 4c,d, the maximum dry density of both soil sample 1 and soil sample 2 decreased with increasing dosages of geopolymer and fly ash. This phenomenon occurred because both geopolymer and fly ash have lower specific gravity. Consequently, as the stabilizer dosage increases, their density exhibits a gradual decreasing trend [32].

This study revealed the differentiated responses of soil sample 1 and soil sample 2 under the action of the fly ash–geopolymer stabilizer. The test results support the classical theory that the stabilizer reduces soil plasticity. However, the conclusion is limited by the single source of the samples, the unoptimized stabilizer ratio, and the lack of long-term performance data. In the future, it is necessary to incorporate various samples to verify universality and optimize the synergy effect of the ratio so as to promote the engineering application of low-carbon soil solidification technology.

### 3.2. Unconfined Compressive Strength Test

**Figure 5:**
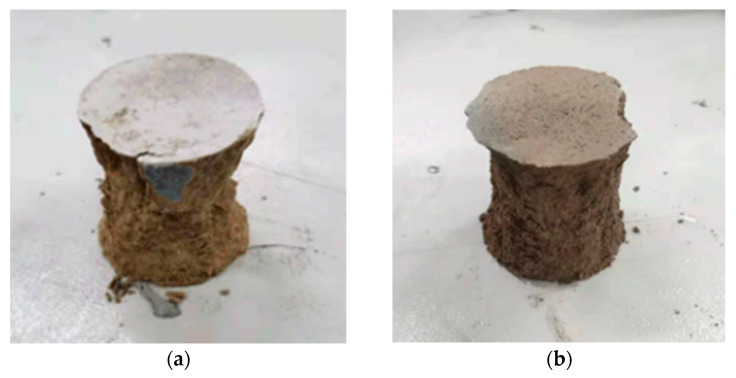
Failure mode of geopolymer-stabilized soil. (**a**) Failure mode of soil sample 1. (**b**) Failure mode of soil sample 2.

Compressive tests were conducted on geopolymer-stabilized soils and geopolymer–fly ash-stabilized soils with varying stabilizer dosages and compared with cement-stabilized soils and cement–fly ash-stabilized soils under identical conditions. Figure 5 illustrates the representative failure modes of geopolymer-stabilized soil sample 1 (left) and soil sample 2 (right) after fly ash incorporation.

#### 3.2.1. Unconfined Compressive Strength of Geopolymer-Stabilized Soil Sample 1

**Figure 6:**
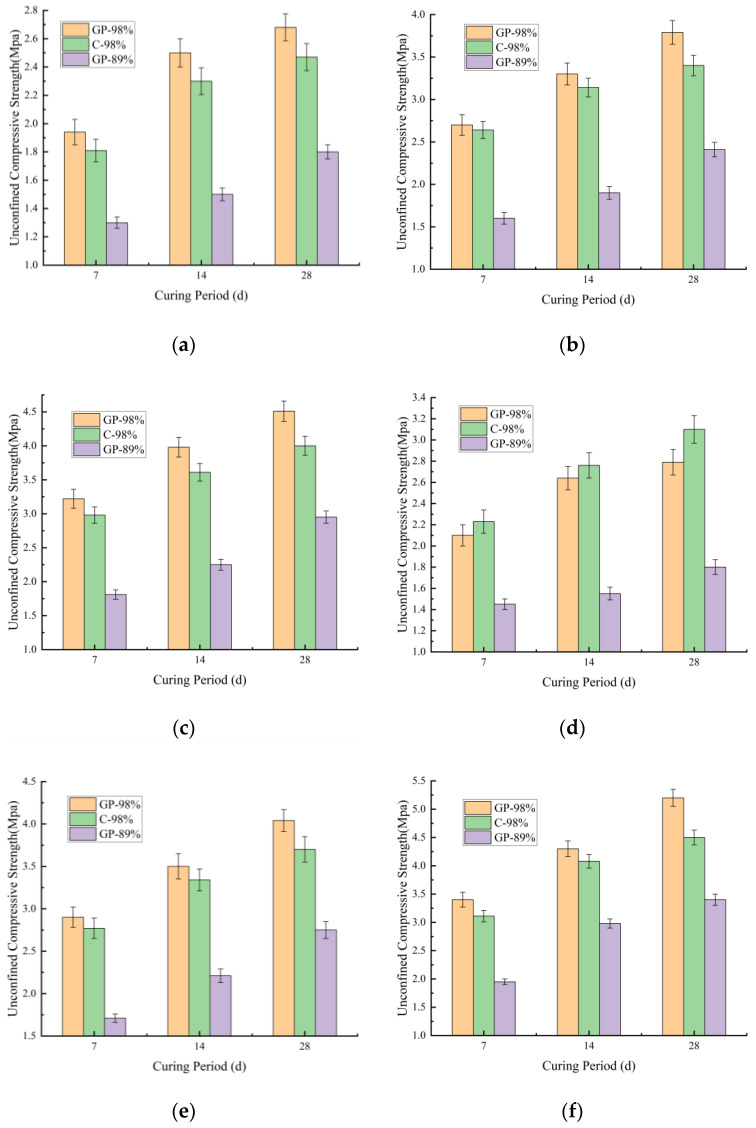
Strength variations of geopolymer-stabilized soil sample 1. The (**a**) 4% geopolymer; (**b**) 6% geopolymer; (**c**) 8% geopolymer; (**d**) 4% geopolymer + 8% fly ash; (**e**) 6% geopolymer + 12% fly ash; (**f**) 8% geopolymer + 16% fly ash.

According to the data in Figure 6a–f, it is readily observable that at a 98% compaction degree, the compressive strength differences between 42.5-grade ordinary Portland cement and geopolymer-stabilized soil sample 1 were generally insignificant. The cement-stabilized soil exhibited marginally lower strength compared to the geopolymer-stabilized variant. This phenomenon originated from both materials’ reliance on dense structural configurations to establish effective cementitious networks. However, geopolymers demonstrated sustained alkaline activation reactions, enabling more comprehensive pore-space filling by cementitious products during later stages. Consequently, they manifested slightly superior strength development potential [33]. As shown in Figure 6a–c, a low dosage of 4% met the requirements for light traffic subbase courses, while higher dosages satisfied specifications for heavy-duty traffic subbase courses or light traffic base courses. At an 89% compaction degree, geopolymer-stabilized soil exhibited significant strength reduction, indicating the high sensitivity of the cementation process to particle compactness. This demonstrated the substantial impact of compaction degree on the compressive strength of geopolymer-stabilized soil sample 1. As shown in Figure 6d–f, following fly ash incorporation, the overall strength trends remained comparable to pre-admixture conditions, except for the group with a 98% compaction degree and a 4:8:88 mass ratio, where geopolymer–fly ash-stabilized soil exhibited marginally lower strength than cement–fly ash-stabilized soil.

The variations of compressive strength with curing age for geopolymer-stabilized soil sample 1 is shown in Figure 6a–f. The figure indicates that the compressive strength of soil sample 1 continuously increased with curing time after geopolymer stabilization, with a notable enhancement from 7 days to 28 days. For geopolymer-stabilized soil at a 98% compaction degree, the compressive strength exceeded 1.9 MPa during the initial curing stage (7 days), which is slightly lower than that of cement-stabilized soil under the same compaction conditions. Compared to the 89% compaction degree, geopolymer-stabilized soil at 98% compaction exhibited significantly higher compressive strength. After incorporating fly ash, the compressive strength of soil sample 1 under geopolymer–fly ash stabilization showed marked improvement. This was attributed to the reactive SiO_2_/Al_2_O_3_ in fly ash supplementing precursor materials for the reaction, while fine particles further filled pore structures [34]. Specifically, geopolymer-stabilized soil at 98% compaction achieved compressive strengths exceeding 2.1 MPa at 7 days and over 1.45 MPa at 89% compaction.

The compressive strength of soil sample 1 increased with the increase in curing time and stabilizer dosage. Zhang et al. [35] discussed the effects of different phases on the mechanical properties of FA/GGBFS-based GPC. Comparisons indicated that FA/GGBFS-based GPC had better mechanical properties than OPCC. The compressive strength of GPC after 28 days of curing can reach 70 Mpa, while that of OPCC after 28 days of curing was close to 60 Mpa. SEM revealed that the structure of the geopolymer was denser than that of OPC. This result is similar to that in this paper, both indicating that slag and fly ash can enhance the mechanical properties of soil or concrete. However, the conclusion was limited by the short-term data of 28 days and did not take into account the fluctuations in temperature, humidity, and economic assessment. In the future, a long-term strength prediction model needs to be established to explore the attenuation mechanism of the structural stability of ground polymers in salting/freeze–thaw environments.

#### 3.2.2. Unconfined Compressive Strength of Geopolymer-Stabilized Soil Sample 2

**Figure 7:**
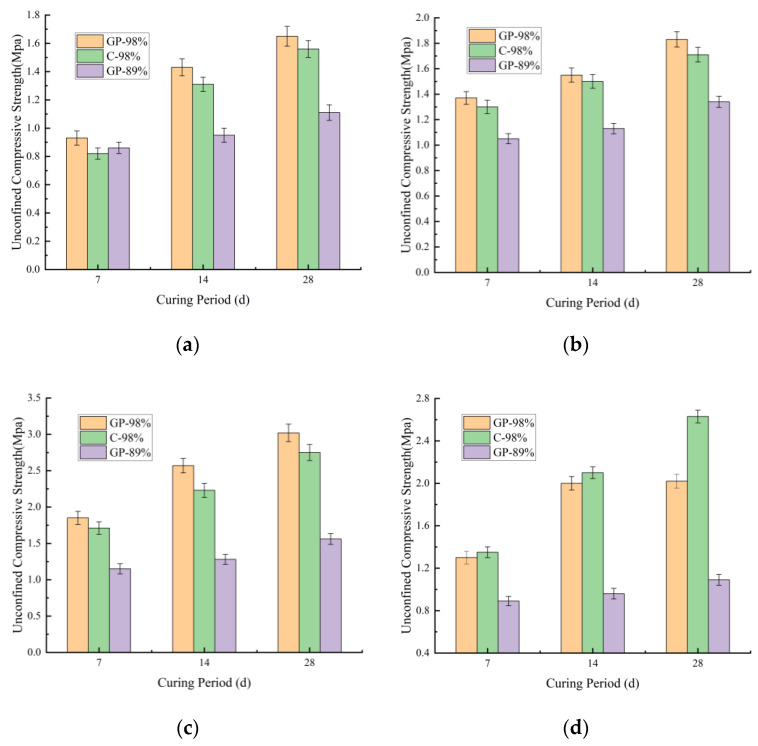

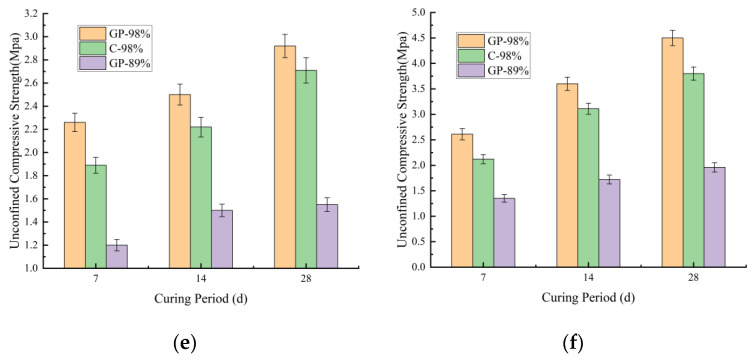
Strength variations of geopolymer-stabilized soil sample 2. The (**a**) 4% geopolymer; (**b**) 6% geopolymer; (**c**) 8% geopolymer; (**d**) 4% geopolymer + 8% fly ash; (**e**) 6% geopolymer + 12% fly ash; (**f**) 8% geopolymer + 16% fly ash.

As shown in Figure 7a–f, the strength behavior of geopolymer-stabilized soil sample 2 aligned with that of soil sample 1. The unconfined compressive strength of stabilized soil sample 2 progressively increased with curing age and compaction degree. However, the stabilized soil sample 2 exhibited relatively low strength values. As shown in Figure 7a–c, at a 98% compaction degree and 4% dosage, the 7-day unconfined compressive strength approximated 0.9 MPa, reaching 1.6 MPa at 28 days. With an 8% dosage, the 7-day strength rose to approximately 1.8 MPa, achieving 3 MPa at 28 days—significantly lower than stabilized soil sample 1. This discrepancy arose from soil sample 2’s higher plasticity index compared to soil sample 1. The sandy soil particles were notably coarser than clay particles, and their morphology displayed more regular geometric features, suggesting a higher skeletal mineral content contributing to structural strength in sandy soil [36]. As shown in Figure 7d–f, after fly ash incorporation, the 4:8:88 mass ratio yielded a 7-day strength of 1.3 MPa and a 28-day strength of 2.02 MPa. The 8:16:76 mass ratio achieved 2.61 MPa at 7 days and 4.5 MPa at 28 days.

At a compaction degree of 89%, it was observed that neither stabilizer dosage variations nor fly ash addition significantly affected the strength of stabilized soil sample 2. When the stabilizer dosage increased from 4% to 8%, the 7-day compressive strength only rose from 0.86 MPa to 1.15 MPa. Upon fly ash incorporation with a modified mass ratio (from 4:8:88 to 8:16:76), the 7-day compressive strength increased from 0.89 MPa to 1.35 MPa.

This indicated the dominant influence of compaction degree on stabilized soil sample 2. This phenomenon was attributed to the high plasticity index of soil sample 2. Under low compaction conditions, the macro-pores formed by its loose structure remained insufficiently compressed. Consequently, both the micro-aggregate effect of fly ash and the activation of reactive components were constrained by discontinuous contact interfaces [37].

#### 3.2.3. Effect of Fly Ash on the Unconfined Compressive Strength of Geopolymer-Stabilized Various Soil Samples

**Figure 8:**
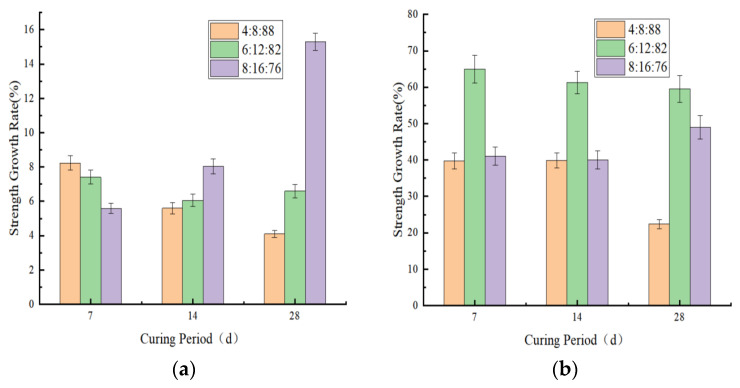
Enhancement rate after fly ash incorporation. (**a**) Strength enhancement rate of soil sample 1. (**b**) Strength enhancement rate of soil sample 2.

Figure 8a,b illustrates the strength growth rates of geopolymer-stabilized soil sample 1 and soil sample 2 with varying stabilizer dosages after incorporating identical proportions of fly ash.

As shown in Figure 8a,b, geopolymer-stabilized soil samples 1 and 2 with varying stabilizer dosages exhibited varying degrees of compressive strength enhancement after incorporating identical fly ash proportions. As shown in Figure 8,b, the compressive strength enhancement rate of geopolymer-stabilized soil sample 2 generally exceeded 40% with fly ash incorporation. Specifically, at 7-day curing with a 6% stabilizer dosage, soil sample 2 demonstrated a strength increase of 65% after fly ash incorporation, whereas soil sample 1 showed a maximum strength improvement of only 15% under the same conditions. These results demonstrated the significantly greater influence of fly ash on the strength enhancement of geopolymer-stabilized soil sample 2.

The mechanism analysis revealed that the higher plasticity index of soil sample 2, combined with the addition of fly ash, which introduced reactive components such as active silica (SiO_2_) and alumina (Al_2_O_3_), facilitated the formation of reactive alumina–oxygen tetrahedra and silica–oxygen tetrahedra under strong alkaline conditions. These precursors underwent polycondensation reactions, initiating gelation within the system. Subsequent reorganization and polymerization processes generated semi-crystalline zeolite-like phases, leading to hardening [38]. This microstructure evolution significantly enhanced the strength and hydrostability while reducing capillary action. Furthermore, the abundant clay minerals in soil sample 2 were synergistically activated by the geopolymer–fly ash system, resulting in substantial compressive strength enhancement. In contrast, soil sample 1 had already achieved high strength through geopolymer stabilization and compaction effects. The subsequent incorporation of fly ash primarily enriched its mineral composition, which contributed to noticeable long-term strength development during later curing stages.

#### 3.2.4. Effect of Compaction Degree on the Unconfined Compressive Strength of Geopolymer-Stabilized Various Soil Samples

**Figure 9:**
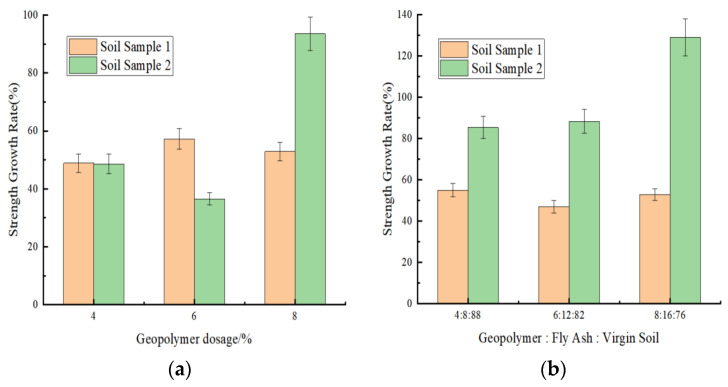
Strength enhancement rate of various stabilized soils with increased compaction degree. (**a**) Geopolymer. (**b**) Geopolymer: fly ash: virgin soil.

By adjusting the compaction degree from 89% to 98%, the 28-day unconfined compressive strength enhancement rates of stabilized soils with varying stabilizer dosages are shown in Figure 9a,b.

As shown in Figure 9a,b, increasing the compaction degree universally enhanced the unconfined compressive strength of all stabilized soils to varying extents. However, the unconfined compressive strength improvement of geopolymer-stabilized soil sample 1 showed minimal sensitivity to stabilizer dosage variations and fly ash addition. Both before and after fly ash incorporation, its strength enhancement rate remained approximately 50%. This observation indicated that the compressive strength of soil sample 2 was predominantly constrained by gelation reaction saturation rather than being solely governed by structural compactness.

For geopolymer-stabilized soil sample 2, however, the unconfined compressive strength enhancement under increased compaction degree exhibited significant dependence on stabilizer dosage and fly ash content. As shown in Figure 9a, when the geopolymer dosage increased from 4% to 8%, the unconfined compressive strength improvement rate of soil sample 2 rose substantially from 48% to 93%. As shown in Figure 9b, with fly ash addition (mix ratio 8:16:76 for geopolymer: fly ash: soil), the unconfined compressive strength of geopolymer–fly ash-stabilized soil sample 2 demonstrated greater sensitivity to compaction degree than soil sample 1. Specifically, increasing compaction from 89% to 98% achieved a 129% strength enhancement rate.

This phenomenon was attributed to the high compaction-induced oriented rearrangement of clay particles, which not only disrupted the physical barriers of the original layered structure but also activated dissolution–polymerization reactions of silicon–aluminum components in fly ash through interparticle contact pressure, forming “micro-regional cementitious nuclei” that reinforced the system. These findings confirmed the critical role of compaction control in optimizing unconfined compressive strength development for both geopolymer-stabilized and geopolymer–fly ash-stabilized soils. Notably, elevating the compaction degree of geopolymer–fly ash-stabilized soil sample 2 yielded disproportionately higher strength gains, compared to other formulations.

### 3.3. Freeze–Thaw Cycle Test

Stabilized soil specimens with a 14-day curing period and a 98% compaction degree were subjected to five freeze–thaw cycles. During the cycling process, surface blistering and spalling occurred, while internal micro-cracks developed due to pore water phase transitions, leading to progressive strength degradation. Specimens with mass loss exceeding 5% were eliminated through weighing. The remaining specimens underwent post-cycling compressive strength testing, and the calculated compressive strength loss (BDR, bearing capacity degradation ratio) results are summarized in Table 4 and Table 5.

As indicated by Table 4 and Table 5, under geopolymer stabilization alone, the bearing capacity degradation ratio (BDR) of soil sample 1 ranged from 67% (minimum) to 78% (maximum), while soil sample 2 exhibited BDR values between 0% (minimum) and 64% (maximum). With combined slag-based geopolymer and fly ash stabilization, the BDR of soil sample 1 increased to 79–90%, whereas soil sample 2 showed BDR values from 0% to 80%. Overall, soil sample 1 demonstrated superior freeze–thaw resistance, compared to soil sample 2, under identical stabilization conditions. Furthermore, under identical mix proportions, specimens stabilized with cement binder exhibited inferior freeze–thaw resistance performance relative to geopolymer-stabilized counterparts.

**Figure 10:**
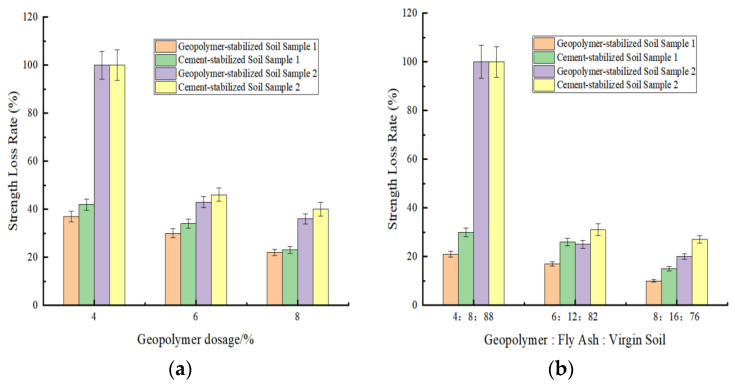
Strength loss rate of various stabilized soils under freeze–thaw cycling. (**a**) Geopolymer. (**b**) Geopolymer: fly ash: virgin soil.

The actual strength loss rate after freeze–thaw cycles was calculated using (1-*BDR*). As shown in Figure 10a,b, the strength loss rate significantly decreased after the incorporation of fly ash. Additionally, the strength loss rate progressively reduced with increasing stabilizer dosage.

This phenomenon primarily stems from the elevated content of reactive mineral components per unit volume, which, under alkaline conditions, promotes the formation of gel phases and zeolite-like phases. These secondary cementitious products densify the internal microstructure by reducing accessible pore structures, thereby enhancing resistance to environmental variations. Aghaeipour et al. [39] studied the effect of GGBS as a cement substitute on the freeze–thaw resistance of RCC at different proportions. The research results showed that after the same number of freeze–thaw cycles, the compressive strength of concrete containing GGBS was greater than that of concrete without GGBS.

**Figure 11:**
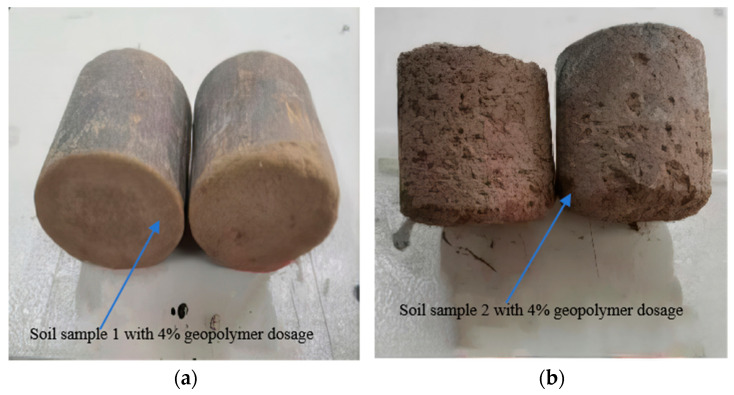
Morphological differences after freeze–thaw cycling (**a**) Soil sample 1 with 4% geopolymer dosage. (**b**) Soil sample 2 with 4% geopolymer dosage.

Visual inspection of post-freeze–thaw-stabilized soil specimens (Figure 11) revealed varying degrees of damage induced by cycling. Severe cases exhibited extensive delamination, geometric distortion, and structural integrity loss. Moderately damaged specimens displayed dense honeycomb-like pitting on surfaces, while minor damage manifested as localized blistering with relatively intact overall morphology.

Comparative analysis indicated that increased stabilizer dosage and fly ash incorporation progressively mitigated frost damage. Cement-stabilized specimens exhibited significantly greater deterioration, compared to geopolymer-modified counterparts. Notably, soil sample 1 maintained structural integrity, even at a 4% stabilizer dosage, without macroscopic spalling, whereas soil sample 2 suffered pronounced damage. This demonstrates that soils with higher plasticity indices exhibit inferior frost resistance, suggesting the preferential use of permeable sandy soils in seasonal freezing regions for engineering applications.

In this paper, polymers based on slag/fly ash were used to replace traditional cement-based binders, and the influence laws on the mechanical strength and freeze–thaw cycle resistance of the stabilized soil base were systematically studied to verify its feasibility as a semi-rigid base material. Meanwhile, high-performance road materials were prepared by utilizing industrial waste residues (such as slag and fly ash), reducing carbon emissions and promoting resource recycling, making contributions to the development of materials science.

## 4. Conclusions

This study systematically investigated the road performance of two distinct soil types stabilized with slag-based geopolymer and fly ash. Key conclusions were as follows:

(1) Slag-based geopolymer significantly enhanced the compressive strength of both soils, with strength positively correlated to dosage. At a 98% compaction degree, soil sample 1 (7-day: 3.4 MPa, 28-day: 5.2 MPa) and soil sample 2 (7-day: 2.61 MPa, 28-day: 4.5 MPa) exhibited higher strength than cement-stabilized soils at equivalent dosages. The stabilized soils can satisfy the strength requirements for subbase layers in extremely heavy/special heavy traffic roads and base courses in light traffic roads.
(2) Fly ash incorporation significantly enhanced the compressive strength of both soils, with more pronounced reinforcement effects on soil sample 2. Under a 98% compaction degree, fly ash addition generally increased the compressive strength enhancement rate of geopolymer-stabilized soil sample 2 by over 40%. Notably, specimens with a 6% stabilizer dosage achieved a 65% strength increase at 7-day curing. In contrast, the maximum strength improvement for soil sample 1 under identical conditions was limited to 15%.
(3) Compaction degree elevation substantially improved the unconfined compressive strength of stabilized soils. The unconfined compressive strength enhancement rate of geopolymer-stabilized soil sample 1 remained stable at approximately 50%, while soil sample 2 achieved a maximum strength increase of 129%, demonstrating significantly higher compaction sensitivity than soil sample 1. Consequently, the comprehensive stabilization of soil sample 2 using geopolymer–fly ash systems required enhanced compaction quality control during rolling operations to ensure target density attainment.
(4) Synergistic effects of stabilizer dosage and fly ash co-stabilization collectively improved freeze–thaw resistance. Soil sample 1 demonstrated superior frost durability (BDR: 78%) compared to soil sample 2 (BDR: 64%). With combined stabilization, their BDR values increased to 90% and 80%, respectively. Although composite stabilization significantly enhanced the frost resistance of soil sample 2, particular attention remains necessary regarding its initial strength and freeze–thaw susceptibility.

## Figures and Tables

**Table 1:** Atterberg limits of soils.

Soil Sample	Liquid Limit	Plastic Limit	Plasticity Index
1	26.7	18.9	7.9
2	46.1	29.1	17

**Table 2:** Chemical compositions of materials (wt.%).

Material	Na_2_O	MgO	Al_2_O_3_	SiO_2_	P_2_O_5_	SO_3_	K_2_O	CaO	TiO_2_	Fe_2_O_3_
Slag	0	3.06	5.10	17.56	0.43	2.65	1.35	63.45	0.39	4.10
Fly ash	0.888	1.08	32.06	53.94	0.64	0.73	2.10	4.11	1.14	4.15
Cement	0.20	1.85	6.34	18.23	0.22	2.41	0.74	65.57	0.295	3.32

**Table 3:** Compaction test results of geopolymer-stabilized soil in slag bases.

Number	Mixing Ratio%				Optimal Moisture Content	Maximum Dry Density
	Geopolymer	Fly Ash	Soil Sample 1	Soil Sample 2		
1	4	0	0	96	16.1	1.881
2	4	0	96	0	10.9	1.952
3	6	0	0	94	16.6	1.872
4	6	0	94	0	11.5	1.934
5	8	0	0	92	15.4	1.863
6	8	0	92	0	11.7	1.926
7	4	8	0	88	12.8	1.895
8	4	8	88	0	12.1	1.933
9	6	12	0	82	15.2	1.852
10	6	12	82	0	12.3	1.892
11	8	16	0	76	15.4	1.818
12	8	16	76	0	12.8	1.855

**Table 4:** Freeze–thaw cycle strength test results of soil sample 1.

Dosage	Geopolymer-Stabilized Soil Sample 1			Cement-Stabilized Soil Sample 1		
	Strength Before Freeze–Thaw/MPa	Strength After Freeze–Thaw/MPa	*BDR*/%	Strength Before Freeze–Thaw/MPa	Strength After Freeze–Thaw/MPa	*BDR*/%
4	2.5	1.67	67	2.32	1.35	58
6	3.3	2.30	70	3.14	2.06	66
8	3.98	3.11	78	3.61	2.76	77
4:8:88	2.64	2.08	79	2.76	1.93	70
6:12:82	3.5	2.89	83	3.34	2.48	74
8:16:76	4.3	3.85	90	4.08	3.45	85

**Table 5:** Freeze–thaw cycle strength test results of soil sample 2.

Dosage	Geopolymer-Stabilized Soil Sample 2			Cement-Stabilized Soil Sample 2		
	Strength Before Freeze–Thaw/MPa	Strength After Freeze–Thaw/MPa	*BDR*/%	Strength Before Freeze–Thaw/MPa	Strength After Freeze–Thaw/MPa	*BDR*/%
4	1.43	/	/	1.31	/	/
6	1.55	0.88	57	1.45	0.78	54
8	2.57	1.64	64	2.23	1.34	60
4:8:88	2	/	/	2.1	/	/
6:12:82	2.5	1.87	75	2.22	1.53	69
8:16:76	3.6	2.88	80	3.11	2.28	73

## Data Availability

The original contributions presented in this study are included in the article. Further inquiries can be directed to the corresponding author.
